# Thanks for inviting me to the party: Virtual poster sessions as a way to connect in a time of disconnection

**DOI:** 10.1002/ece3.6756

**Published:** 2020-09-14

**Authors:** Emily A. Holt, Ashley B. Heim, Erin Tessens, Robert Walker

**Affiliations:** ^1^ School of Biological Sciences University of Northern Colorado Greeley Colorado USA

**Keywords:** ecology, Mozilla Hubs, online teaching and learning, undergraduates, virtual poster session

## Abstract

COVID‐19 presented the world with trauma and isolation, but many people, including educators, have offered bright spots of creativity and engagement. As we confronted these issues in our own ecology classroom, we sought solutions to carry‐forward the learning objectives we set for our students in January 2020, yet encourage interaction with the sensitivity that a pandemic requires. In the rapid transition to online course delivery, we opted to retain the original end‐of‐semester poster project in our introductory ecology course. However, we experimented with a new virtual platform where students could disseminate their work and communicate with the community. In this paper, we discuss the Mozilla Hubs virtual reality platform that we used for our event. We also collected qualitative data to share the benefits and challenges of this experience felt by the students, the instructors, and external observers.

## Graphical abstract

In the rapid online transition of an introductory ecology course due to COVID‐19, we shifted an end‐of‐semester poster project to a virtual poster session. In this paper, we discuss the Mozilla Hubs virtual reality platform that we used for our event. We also reflect using qualitative data to describe the benefits and challenges of this experience felt by the students, the instructors, and external observers.

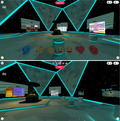

## 1 BACKGROUND

In many ecology classrooms, examinations, written reports, and oral presentations are common assessment methods. However, translating original research projects or reviews of the literature into a culminating professional‐style poster session is increasingly more common as a pedagogical tool (Adkins & Lyons, 2012; Altintas, Suer, Sari, & Ulker, 2014; Hay & Thomas, 1999). The value of these experiences and this assessment model is multifaceted. The projects upon which the posters are based are often collaborative, which contributes to students' development of team‐work skills (Mulnix & Penhale, 1997) and lowers the grading burden of the instructor with fewer total assignments to evaluate (Hess & Brooks, 1998). The final outcome represents a concrete end product (Stewart, 2008) and can provide a creative outlet for students in the sciences (Wimpfheimer, 2004).

Creating posters is considered an effective path to immersing students in complex and sometimes controversial topics (Deutch, 2011; Dorner, 2015). Consolidating all the relevant findings into a concise and attractive poster, further hones synthesis skills (Kinikin & Hench, 2012) and graphic communication (Sweeney, 1984). Posters represent a unique communication medium where the poster itself could communicate the message alone, but the attending presenter provides a mode of two‐way interaction to delve more deeply into the content (MacIntosh‐Murray, 2007). The presentation of posters by students fosters skills in oral communication (Chan, 2011) and science communication (Mayfield, Olimpo, Floyd, & Greenbaum, 2018). Participation in a public session allows students to further exercise these skills with nonscience members of their community and engage in an authentic scientific task of disseminating their work (Baumgartner, 2004; Mulnix & Penhale, 1997).

Despite the noted benefits, most reports of class‐based poster sessions are in‐person experiences, and proposed online alternatives are neither interactive nor synchronous (Kinikin & Hench, 2012), failing to provide students with real‐time feedback. However, online education is ubiquitous; one in four students will take at least one online course in their undergraduate degree program, and one in seven students in higher education exclusively takes online classes (Allen & Seaman, 2016). COVID‐19 may forever change the instructional landscape where online alternatives are critical, not exceptional (cite).

In this paper, we report on a novel, virtual venue to hold course‐based poster sessions. In past years, our ecology course has successfully used posters as a key assessment. We normally held a formal poster session, where each student group's posters were professionally printed and displayed on large boards, which was open to the public the last day of each semester. Given the abrupt mid‐semester transition online, our instructional team shifted our session into a new virtual format. We hope by describing the experiences of our students, external observers, and ourselves as instructors that this format may be available to a greater number of pedagogical applications as instruction increasingly shifts online.

## 2 CLASS AND PROJECT DESCRIPTION

Students (*n* = 66) in an introductory, upper‐level ecology course were required to participate in a small group research project that resulted in a poster reporting on their work. Originally, all pieces of this assignment represented 25% of their final grade, which included a written proposal describing their research plan and a poster to disseminate their findings. With guidance from their teaching assistant, students were expected to conduct novel research projects where they devised original research related to ecology, developed an appropriate study design, collected and analyzed their own data, and reported on their findings as a group. By the ninth week of a 16‐week semester, students had already begun to compile relevant literature related to their self‐selected topics and formulate a hypothesis to investigate their research question, and all had developed a protocol to test their hypotheses. While most students were in preliminary phases of data collection, none had collected all of their data, and further, some had not yet started on data collection. Projects they developed were primarily laboratory experiments (e.g. effects of predator type on aphid abundance, effect of bisphenol‐A on *Bouteloua gracilis* vegetative growth, effect of microplastic abundance on clam behavior) with one observational field study (water quality surveys above and below urban centers along a nearby river).

In light of the COVID‐19 pandemic, most classes at institutions across the United States were shifted swiftly and entirely to an online learning environment. Unfortunately, in our ecology class, none of the students' research projects could be salvaged because each would have required in‐person monitoring of data collection. To address some of our original objectives (i.e. analyze data, summarize findings, and present their work; Appendix S1), we, as the course instructors, remodeled the assignment given the constraints. The outcome would remain unchanged (i.e. a poster to be presented to a public audience); however, the project became a literature review and position poster (Dorner, 2015) on an assigned ecological topic. We opted to randomly assign topics rather than allow students to choose topics as we did at the start of the semester to expedite their progress. Through this revised project, students were expected to examine an ecological issue and its relationship to humans by assembling evidence, formulating a thesis, and presenting an argument (Appendix S1). Our challenge was finding a new venue that simultaneously supported social distancing and brought together presenters and a local audience.

## 3 MOZILLA HUBS PLATFORM

We opted to use the open source Mozilla Hubs virtual reality (VR) platform. In exploring potential venue options, we found that an international conference on VR applications (http://ieeevr.org/2020/online/) was providing a remote environment for synchronous paper and poster presentations using this platform. Hubs is a social multiuser VR chat room that is accessible from any browser, introduced in 2018 (https://hubs.mozilla.com). Hubs is open source and customizable through its sister platform, Spoke, an online 3D scene editor. We chose Hubs because it offered the greatest capacity for a shared inclusive learning environment. Hubs works on any device (i.e. desktop computer, laptop, tablet, mobile device) and supports all VR hardware but also accommodates devices without these upgrades. It is also accessible through any browser, further extending its access to all students.

**FIGURE 1:**
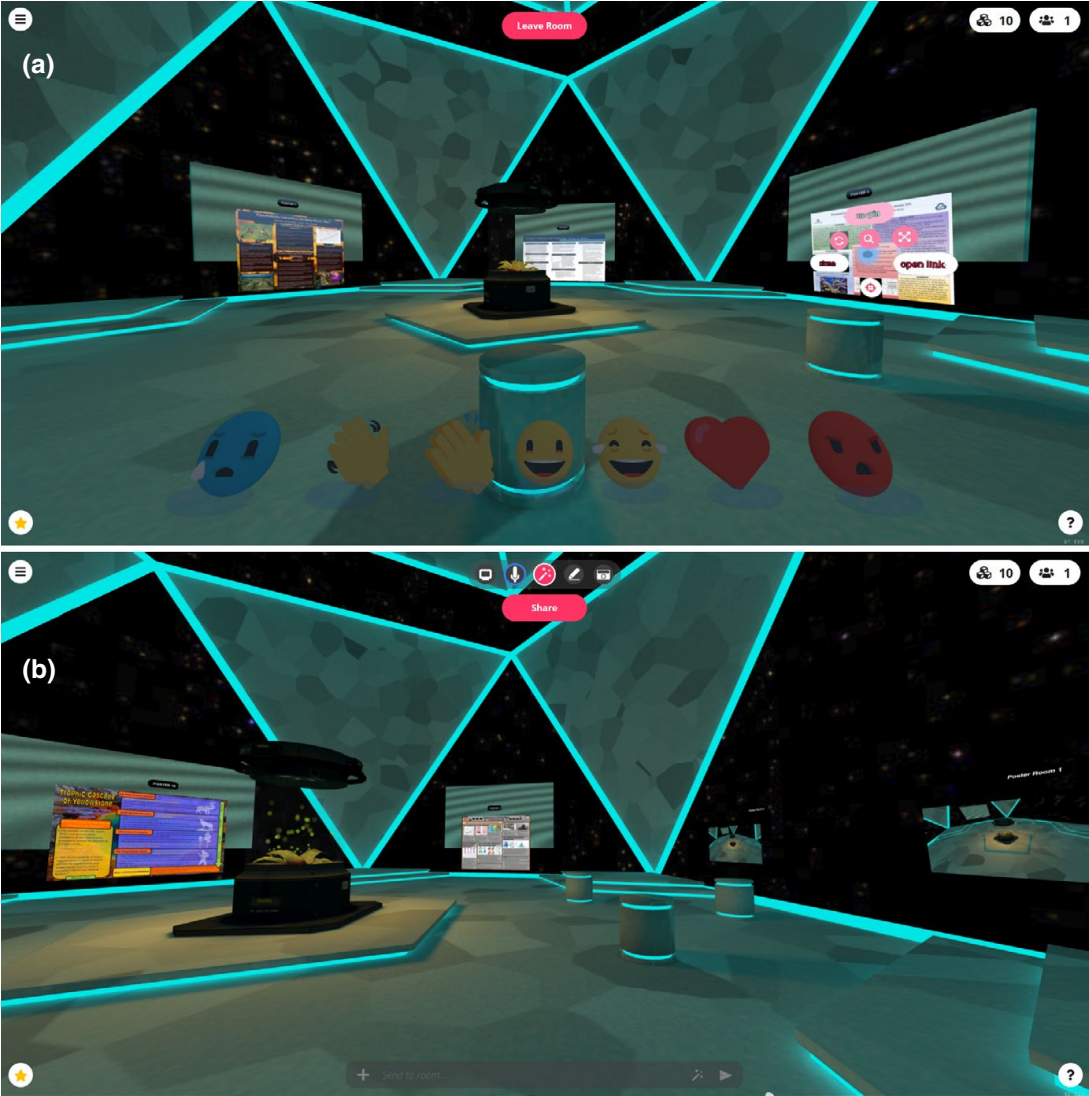
Mozilla Hubs ecology poster session rooms. (a) View of one of the presentation rooms upon entry, showing relative arrangement of three student posters in the distance. In the poster on the right, a user has activated menus to investigate objects that appear as pink circles or text (e.g. zoom closer to poster text, open in a separate browser tab). At the bottom of the screen, a user can use emojis to respond to what they see in the virtual space. (b) View of another presentation room, showing “doorways” to neighboring rooms, which are the two floating objects to the right. Attendees are represented as customizable avatars (not pictured) and move freely around the space using keypad/mouse, touchscreen, or wired‐in headset to interact with other users and objects. Attendees communicate through audio and a chat

We decided to have all attendees and presenters enter an atrium as a single entry point into our session. This scene had six one‐way links to the main presentation rooms from the atrium. Presentation rooms were linked in a ring structure with two‐way links connecting each room to the next and previous room (Appendix S2). Each room looked identical, except several walls housed a poster number under which.jpeg images of posters were placed (Figure 1). We limited three posters per room, as the Hubs‐suggested user limit for each room was 24 people, and each of our posters had 3–5 presenters that rotated through presenting. Beyond 24 users, attendees would be held in a waiting area for each room until a user left. Since we were uncertain how many attendees would come, we opted for more rooms housing fewer posters to give us greater flexibility and lessen the VR burden on participating devices.

Students worked in groups of one to five individuals on their collaborative posters, and each was preassigned a minimum of half‐hour blocks to “stand by their poster” to synchronously interact with any interested viewers (i.e. peers or nonpeers) during a two‐hour synchronous poster session. In each of six virtual presentation rooms, presenters stood next to their own posters and faced their avatars toward the center of the room. Hubs sound quality mimics a live space where avatars close to you sound louder and those further away are quieter. Additionally, when you turn your own avatar, the sound “changes based on directionality.” Everyone's audio is shared in a Hubs room similar to a live experience, where background conversations exist but immediate discussions are clear. Attendees also had the option to use a text‐based group chat function (Figure 1).

The use of Mozilla Hubs as an educational platform has been sparsely reported, which may simply be a relic of its relative novelty. Relevant studies tend to focus on (a) the technological development of Mozilla Hubs for learning purposes rather than on the application of course curriculum using Mozilla Hubs (Scavarelli, Arya, & Teather, 2019a, 2019b) and (b) comparing features across VR platforms and advising which platform may be most effective for educational purposes (Harfouche & Nakhle, 2020; Scavarelli, Arya, & Teather, 2020).

## 4 DATA COLLECTION

The procedures for this study were approved by the Institutional Review Board of the University of Northern Colorado (IRB #2005001956). Verbal or written consent was obtained by all participating external observers. As we collected student reflections, that were an assignment as part of the class, retroactively, consent from student participants was not necessary to obtain.

### 4.1 Instructor and external observer reflections

We invited five instructors who were not associated with the course (*n* = 3 within the same institution, *n* = 2 outside the institution), but who participated in the session real‐time, to provide their reflection as external observers. Additionally, the four instructors of the class also provided their insights into the poster session. Each of the authors (henceforth called instructors) individually responded to a set of reflection questions that primarily focused on the process of poster preparation, disseminating research at the poster session, and the benefits and challenges of the poster session from both an instructor's and student's point of view (Appendix S3). We then discussed our individual responses as a group to collaboratively identify common themes and highlight unique experiences. We provided the same set of reflection questions (Appendix S3) to our external observers and received feedback from three of them. We used reflections from the external observers to supplement the themes we discussed as instructors.

### 4.2 Student reflections

Within 5 days of the poster session, students were required to provide written responses to the following reflection questions as part of a poster session evaluation assignment via our course learning management system (LMS): (a) reflecting on our ecology virtual poster session, what surprised you the most in this experience? (b) Reflecting on our ecology virtual poster session, what were the greatest benefits of this experience? (c) Reflecting on our ecology virtual poster session, what were the greatest challenges of this experience? And (d) think of past times that you have presented your own work (e.g. project in a class, undergraduate research). How did our ecology virtual poster session compare to that experience?

We used thematic analysis to analyze student responses to the virtual poster session reflection questions. We inductively coded responses into naturally emerging themes (Aronson, 1994) using NVivo 12 (QSR International, 2020). We collected a complete set of responses from 63 of 66 total students enrolled in the course.

Individually, a single researcher completed an initial coding of the open‐response student data. Then, two researchers came to a consensus on final themes with high intercoder agreement for each reflection question (Creswell, 2013). We separated all challenges and benefits by whether they were associated with the virtual poster session itself or with project preparation for the session. We do not report on the latter, which mainly focused on creating the poster, group work, and workload considerations.

## 5 FINDINGS OF PERCEIVED BENEFITS OF THE VIRTUAL POSTER SESSION

Both students and instructors perceived several benefits or successes of the virtual poster session, despite anxieties leading up to the event. We coded student responses into benefits applicable to any poster presentation environment or benefits applicable only to the virtual poster presentation environment. Student perceptions of benefits are then followed by a summary of our perceptions of benefits as instructors supplemented by those of external observers. Total number of references for each theme, which mostly equated to the number of students with a given response excepting for students who cited a single theme multiple times, is noted in parentheses below.

### 5.1 Student‐perceived benefits applicable to any environment

Students cited multiple benefits of ecology poster sessions in general. The most commonly cited benefit was learning ecological topics (*n* = 27), which encompassed learning about topics other than an individual's assigned research topic, learning about topics that one was assigned to research, and teaching others about ecological topics during the session. Other studies likewise suggest poster sessions can serve this heuristic function (Adkins & Lyons, 2012; Rowe & Ilic, 2009). For example, one student mentioned: “What surprised me the most [during the poster session] was probably how many of these topics in ecology are anthropogenically induced. It was an insightful experience overall.” The second most frequently cited general benefit was being able to interact with and present research to others at the poster session (*n* = 21), including peers, instructors, and those outside of the course. Lastly, students also cited flexibility as a general benefit, noting that they were able to develop science communication and public speaking skills (*n* = 3). One student explained: “[The poster session] actually helped me confidence‐wise to express the knowledge I have gathered through this course and better prepare me for future projects.”

### 5.2 Student‐perceived benefits specific to virtual environment

Students also cited multiple benefits unique to virtual poster sessions. The most commonly cited benefit was flexibility (*n* = 24), which encompassed science communication and public speaking skills specific to the virtual environment, navigation or moving around the virtual Mozilla Hubs space, and time management skills. Students felt that there was “less pressure and stress” associated with presenting in the virtual environment, as they were able to discuss their poster with others as an avatar from the comfort of their homes. Navigation‐wise, students believed it was easy to move between virtual rooms in Hubs to view their peers' posters. As each group member was required to present at their poster for approximately thirty minutes, students expressed that the poster session required them to keep track of time more efficiently since we as instructors could not circulate to all attendees and provide frequent time updates.

Further, students commonly referred to the novelty of the virtual poster session and becoming more comfortable in an online platform as a benefit (*n* = 20). Many thought the event was “interesting” and “cool” and enjoyed the Hubs platform as a means of presenting posters. Similar to the instructors, few students seemed to know the existence and capabilities of this online platform prior to this course.

Finally, students described interactions in the virtual environment as an additional benefit (*n* = 14), particularly focusing on how avatars allowed for authentic communication with others (which many students seemingly did not expect). As one student discussed: “I think the greatest benefit was that it allowed for us to interact with our audience members as we would have if the poster session was in person. It was kind of cool to stand by our virtual poster and see the avatars looking at our poster as actual people would have.”

### 5.3 Instructor and external observer‐perceived benefits

We as the course instructors generally felt that students achieved the original learning objectives of the ecology poster session, based on student reflections and anecdotal communications. One primary benefit that we perceived was the sense of community that this virtual poster session established; students were able to interact not only with us and their peers, but were also able to discuss their research with faculty and students outside of the ecology course. An external observer mentioned, “overhearing students chatting and socializing as they usually do at in‐person poster sessions”. Further, paralleling student perceptions, we thought more people were able to attend the poster session because it was online. The virtual nature of our poster session could potentially reach a larger and more diverse audience across institutional and state boundaries. Rather than physically navigating to a location on campus—which would essentially limit attendees to those at our institution—we were able to invite outside faculty and students to join our session from the comfort of their homes and potentially just “drop in” for a short interaction.

Due to the diverse attendees, ease of avatar‐based communication, and organization of Mozilla Hubs, we felt that the virtual poster session mimicked an authentic face‐to‐face poster session. Many students that had previously given face‐to‐face presentations at professional conferences or in other courses also voiced similar sentiments; for example, one student cited: “The [virtual] experience felt very similar [to a face‐to‐face presentation] since you could see someone standing in front of your poster and hear them like they were actually there.”

We also perceived improved engagement among students in the virtual poster session. As students were presenting as avatars, they appeared more comfortable communicating with peers, instructors, and those outside of our course that they had not previously met in person (e.g., faculty from other institutions, students from other classes on campus, etc.). Interestingly, we also observed that students tended to travel between poster rooms and view posters in social groups—often groups that they collaborated with during our face‐to‐face classrooms; thus, the virtual poster session seemed to encourage rather than limit peer‐to‐peer interactions. One benefit that we did not consider prior to the virtual poster session was that avatars allow for a “guise of engagement”; even if a student presenter was bored or distracted while awaiting an audience, avatars always appear attentive and ready to engage, potentially inviting more interaction from other attendees. Similarly, avatars shielded attendees from the awkward interactions that inevitably occur during face‐to‐face poster sessions (e.g., making eye contact with a presenter whose poster topic you are not interested in).

## 6 FINDINGS OF PERCEIVED CHALLENGES OF THE VIRTUAL POSTER SESSION

Similar to the perception of benefit of this novel virtual poster session, both instructors and students identified challenges. Some of these challenges were unique to the virtual environment, while others were challenges of any poster session and students likely noted them due to their inexperience with poster sessions in general. Total number of references for each theme, which mostly equated to the number of students with a given response excepting for students who cited a single theme multiple times, are noted in parentheses below.

### 6.1 Student‐perceived challenges applicable to any environment

The single greatest challenge (*n* = 15) noted by students was communicating in a poster session environment. Eighty percent of these references referred to students' frustration with the “background noise of other presenters” that they felt was distracting. However, the noise level was remarkably similar to a real poster session, which undeniably gets louder as more people engage in their own interactions side by side. Other noted communication challenges related to students' inexperience in presenting (e.g. how to pitch their topic to audiences of different levels of understanding, whether they should deliver “everyone an individual presentation or not”), and these challenges would likely feel burdensome regardless of the online versus face‐to‐face environment. The only other challenge mentioned by students (*n* = 4) that we noted would occur regardless of the environment was the “lack of people that actually came past our poster”. The inherent flexibility of poster sessions (Maugh, 1974) allows attendees to congregate around posters of interest or by presenters they know. This bias, unfortunately, leaves other presenters without an audience. In a virtual environment, this problem could be better mitigated by balancing the number of attendees with the possible rooms to keep the rooms closer to their capacity so that few student presenters get forgotten.

### 6.2 Student‐perceived challenges specific to virtual environment

The students in our sample also noted challenges that were specific to the virtual environment. For example, the challenge of presenting was further complicated by being online (*n* = 15). “Interacting without body language and facial expressions was weird” and “difficult”. Students voiced concern that without this feedback, they were unsure if “[their] message was being delivered [effectively]”. However, the most common challenge (*n* = 39) was technical difficulties related to problems with internet connectivity, their devices, or navigating within the Mozilla Hubs program itself. The single greatest complaint (*n* = 18) was issues with audio. No student reported that they had no sound; rather, they admitted that their sound was “bad quality” or that it “would cut out” leading to a “verbal and virtual tango.” These concerns may have been overstated because, as part of the survey, students were forced to identify challenges and a smaller percent than the total number of references identified explicitly that they personally experienced these challenges.

### 6.3 Instructor and external observer‐perceived challenges

We noted some of the same challenges voiced by our students and experienced a few instructor‐specific challenges. The first set of challenges centered around planning and preparation for the virtual event. The original event had been planned as an on‐campus event, and the space and poster boards were rented prior to the start of the semester. Given the rapid transition online due to the pandemic, we had to redefine the session. We considered several alternatives (e.g. synchronous Zoom presentations, asynchronous posters available on our LMS, virtual session via Discord), before deciding on Mozilla Hubs. Since this was a new platform for all instructors and students, a major challenge was anxiety with an unknown and “not being able to envision what [the session] would look like”. Many of the “unknowns” associated with the virtual poster session also led students to worry about how their poster presentations would be graded; uncertainty about the virtual environment seemed to encourage higher grade motivation among students. In retrospect, these concerns manifested only as perceived challenges, not actual challenges, as many students (*n* = 8) directly commented that they were “surprised how well it worked” and “how smoothly it went”. Interestingly, leading up to the session many students expressed anxiety about presenting as avatars to other avatars—concerned about the quality of online communication and their ability to recognize those to whom they were presenting (e.g. an introductory biology student or the department chair). Ironically during the session, it was the avatars that became “characters behind which the students could hide to ease anxiety about presenting” in a public but classroom environment. Future research could investigate whether virtual poster sessions alleviate student anxiety about public speaking and create more inclusive environments.

As an additional instructional burden, beyond fielding the usual “poster session etiquette” questions in advance of the session, we wanted to master Hubs ourselves to properly train our students on the platform. During the session, instructors, and external observers noted a few additional challenges. While not a rampant issue, we did notice several students experiencing difficulty with their sound and it was a challenge to troubleshoot and assure them that technical difficulties would not negatively impact their grade. An external observer, who visited the session with no training, entered the session and “walked into a wall and stared at a ceiling for 5 min while [she] learned how to turn around,” thus inexperience with gaming and VR navigation could be a hindrance to faculty or students without training or an interest to learn.

## 7 THREE KEYS FOR SUCCESS

We received resoundingly positive feedback from instructors, students, and external observers about this novel ecology course‐based assessment of learning. In reflection, we felt three factors were key in our success.

First, Mozilla Hubs is an open source VR platform, and users can build their own scene in Spoke, involving a mind‐boggling number of choices from details of the objects in a scene, the height of the agent (i.e. avatar), and the position of the spawn point where new visitors enter the scene, just to name a few. As ecologists and not computer scientists, we were fortunate that the community at Hubs was exceptionally friendly and helpful, and one of their research scientists had written a blog post detailing how to run a poster session in their platform (https://blairmacintyre.me/2020/04/10/doing-a-poster-session-in-hubs/). Nested within his post was the prebuilt scene, which optimized the sound for multiple small groups within a single space. While our students complained about hearing other presenters and conversations, ironically, this detail contributed to the authenticity of a real poster session experience noted by instructors and external observers who are regular conference attendees.

Second, two members of our instructional team created adjoining practice Hubs rooms and a handout describing the session layout and Hubs navigation (Appendix S2). Teaching assistants held weekly, synchronous lab check‐ins with individual groups following the online transition via Zoom. However, the final check‐in was hosted within the practice rooms themselves. The goal was to provide a vision for the session, and force students to practice navigation and communication in the virtual environment ahead of the actual session. We feel that the handout combined with this prior exposure to the platform was critical to the success of the session.

Third, the Hubs platform granted our team the control to manipulate our own space while limiting that of outside users. Hubs provides a shared space where instructors, when assigned as moderators, could collaboratively create, move, and resize media in the virtual space. Moderators also had the capacity to remove disruptive attendees. The collaborative nature helped our team build the space and test flaws together (e.g.,.pdf files of posters would not load on mobile devices but.jpeg files were viewable on all devices; file sizes needed to be < 4,000 KB or else they would not load properly). However, we also disabled creating objects, drawings, and emojis for all regular users through Room Settings, which minimized intentional or unintentional disruption of the session space.

## 8 OTHER CONSIDERATIONS FOR FUTURE CHANGE

Having the experience of hosting a virtual class‐based poster session in Mozilla Hubs, we can also reflect on three suggestions for future educators hoping to use this format as an assessment in their own class. First, technical difficulties, namely audio issues, were an oft‐cited limitation of this virtual format. While we advised all presenters and attendees use fully charged devices and close all background applications, perhaps additional training on troubleshooting audio issues might have reduced student anxiety and prepared them to be more capable to resolve issues real‐time and engage more fully.

Second, every Hubs user has the capacity to select their own avatar upon entry. While this choice shares some control and creativity with the community participating, we noticed some distracting selection of avatars. Specifically, one student presenter unknowingly selected an oversized avatar who occupied too much space in each room and obscured posters from viewers standing behind her. Future sessions might benefit from additional guidelines provided to attendees on an appropriate selection of avatars to better support the experience by all attendees.

Third, we invited several local and remote colleagues as well as promoted attendance and engagement with presenters as an extra credit opportunity for our local introductory nonmajors biology class and an upper division ecology course. We provided these people with the weblink to the atrium room, or rather a weblink to a Google doc that housed the Hubs link, where we could time the opening of our Atrium until the posters were set up and we were ready to begin the event. Boasting a privacy‐centric virtual space, Mozilla does not collect nor store any personal information (i.e. there is no login or sign‐up required if you have an invitation link). However, following the session, we realized that it might have been nice to have an estimate of total attendance and a record of outside students to provide their instructors to award extra credit for participation. Future applications that need to track room analytics could require registration through the Hubs Discord Bot (i.e. a mechanism to associate a Hubs room with Discord, a popular gaming chat server).

## 9 ADDITIONAL POSSIBILITIES FOR FUTURE VIRTUAL POSTER SESSIONS

Many societies had to adapt their spring and summer 2020 conference agendas to respond to travel restrictions and minimal social gatherings. The 2020 Ecological Society of America meeting hosted posters that are not interactive (i.e. only asynchronous question‐and‐answer options), the 2020 Evolution meeting canceled all its contributed poster and paper presentations, and Botany 2020 and the Society for the Advancement of Biology Education Research 2020 annual meetings both held online poster sessions where pdf files of posters were shared and the audience could synchronously ask questions in Zoom meeting. While poster sessions are ubiquitous for dissemination at scientific conferences, they represent a growing avenue to assess student synthesis and presentation of class‐based outcomes (Altintas et al., 2014). Adkins and Lyons (2012) further suggest that poster sessions are a viable mechanism to educate the local community about scholarly topics. The benefits of posters as an assessment are numerous, and shifting them into a virtual setting helps reduce many of the drawbacks associated with in‐person sessions. Virtual sessions lack financial costs associated with printing (Baumgartner, 2004) and reserving a large local space, lack transport of fragile posters to the session space (Stewart, 2008), and involve no postsession clean up.

Additionally, learning is a social and collaborative process and interactive sessions allow for reciprocal teaching (Stewart, 2008). In online environments, there is growing interest in identifying avenues to engage students in more intimate small group formats (Cherney, Fetherston, & Johnsen, 2018). One of our external observers noted that our virtual poster session built a platform where students could “have a moment of commiseration of stresses and failures which is a big part of building a sense of belonging for them” which she noted would have otherwise been absent if poster presentations had simply been prerecorded.

While we had success using Mozilla Hubs as our platform, there exists a growing number of similar platforms with varying capacities (Harfouche & Nakhle, 2020). Further, virtual poster sessions are just one of the multitude of pedagogical tools to engage and interact with students remotely (Knapp, 2018). We also envision that the flexibility of a virtual environment would allow collaboration across institutions where students could participate in a poster session representing many different universities.

## 10 CONCLUSIONS

Students, instructors, and external observers all perceived several benefits and challenges of a virtual poster session. The key benefits of a poster session identified by students were learning ecology and interacting with peers; specific to the virtual environment, students appreciated the flexibility, novelty, and the ability to interact in an otherwise isolated COVID world. Instructors and external observers noted that students engaged with each other and the content to become part of a community. We further felt this remote venue did not detract from the virtual experience as it felt authentic to a professional session. To oppose the perception of benefits of this virtual poster session, students, instructors and external observers also noted several challenges. Student concerns primarily centered around technical difficulties with their Internet, device, or the software, and while these challenges were also observed or experienced by instructors or external observers, it seemed easily remedied by more practice. Prior to the event, many students anecdotally expressed apprehension with simply presenting posters, communicating effectively with avatars, and the logistics of the virtual environment. Most of this anxiety was dispelled by practice sessions with the teaching assistants the week before the event.

Past literature suggests that posters can be an effective assessment of a number of important scientific skills. Further, presentation of course‐based posters in a public session provides interactive opportunities beyond those of their near‐peers, allowing communication to a broad audience. One of our external observers reflected on her own formative experience:

“as a student [presenting in a poster session] was the most nerve wracking experience [but] in retrospect it was one of the more informative and encouraging experiences that led me to consider a research path.” In an uncertain time where online education will certainly play an integral role in teaching undergraduate ecology and beyond, we hope educators will be creative to retain these influential experiences in online environments.

## CONFLICT OF INTEREST

None declared.

## AUTHOR CONTRIBUTIONS

**Emily A. Holt:** Conceptualization (lead); data curation (equal); formal analysis (equal); writing – original draft (equal); writing – review and editing (equal). **Ashley B. Heim:** Conceptualization (supporting); data curation (equal); formal analysis (equal); writing – original draft (equal); writing – review and editing (equal). **Erin Tessens:** Conceptualization (supporting); writing – review and editing (equal). **Robert Walker:** Conceptualization (supporting); writing – review and editing (equal).

## Supporting information

Appendix S1

Appendix S2

Appendix S3

## Data Availability

De‐identified data: Dryad https://doi.org/10.5061/dryad.qfttdz0f8.
