# Supplementary material for: Thanks for inviting me to the party: Virtual poster sessions as a way to connect in a time of disconnection
Source: Ecol Evol. 2020 Sep 14;10(22):12423–30. doi: 10.1002/ece3.6756 (PMC7679537; doi:10.1002/ece3.6756)
Supplement: Supplementary file 1 — Appendix S1 [file ECE3-10-12423-s001.docx]

**Appendix S1. Learning objectives and grading rubric for virtual poster session**

**LEARNING OBJECTIVES**

At the conclusion of this COURSE (as a whole), you should be able to…

1. Recall and assemble facts and concepts of ecology.
2. Explain and model how organisms interact with one another and their environment.
3. Apply ecological principles to natural populations and communities to answer ecological and environmental questions.
4. Analyze, interpret, and create graphical data to represent ecological processes.
5. Recognize plagiarism and demonstrate your ability to avoid it through adequate paraphrasing, quoting, and citations in all assignments.
6. Summarize scientific literature to inform a novel research study.
7. Use the scientific method to devise a research project to study an ecological question.
8. Prepare and present a poster as a group to your peers and the UNC community.

By the end of the POSTER PROJECT, students should be able to…

1. Examine an ecological issue and its relationship to humans: assemble evidence, formulate a thesis, and present an argument.
2. Prepare a scientific quality poster on your topic.
3. Argue your thesis in a well-informed and persuasive manner during a poster session.
4. Judge and assemble information presented by your classmates’ posters.

**VIRTUAL POSTER PRESENTATION GRADING RUBRIC**

| **Score** | **7 - 6** | **5 - 4** | **3 - 2** | **1 - 0** |
| --- | --- | --- | --- | --- |
| **Writing:**  **Readability**  **Font**  **Mechanics** | Easy to read without zooming too far. Font size, color and type contrast well with background for optimal readability. Text well written, clear and concise. | Font choice mostly appropriate but perhaps inconsistent throughout poster. Text well written but overly lengthy. | Font choice not optimal but still readable. Text adequately written and reasonably easy to follow. | Difficult to read without zooming in a lot, or barely enough information to fill the poster. Font distracting or illegible. Text poorly written and hard to follow. |
| **Organization:**  **Writing**  **Sectioning**  **Use of visuals** | Obvious defined sections with clear, relevant headings. Excellent and creative use of visuals to enhance the information. | Sections with vague or misleading headings. Good use of visuals. | Poster sectioned without headings. Some use of visuals. | Lack of sectioning makes poster difficult to read. Little use of visuals to convey information. |
| **Content:**  **Thesis**  **Details** | Thesis well developed and effectively supported. High level of relevant evidence presented, yet not so elaborate so as to become tedious. Clearly and effectively presents both sides of the issue. | Thesis is evident with some supporting evidence. Sufficient detail provided to inform the viewer. Provides both sides of the issue, but is more biased towards one side. | Thesis is not developed, with absent or vague supporting evidence. Some information was too detailed or was lacking entirely. Provides few details of opposing side. | Thesis poorly developed. Technical details lacking or inadequate to inform the viewer. No evidence, just unsupported facts. Provides no evidence on the opposing side of the issue.. |
| **Presentation:**  **Knowledge**  **Response** | Presenters demonstrate full knowledge of the material and can explain and elaborate on questions from viewers. | Presenters have sufficient knowledge of the material to answer questions posed by viewers. | Presenters have difficulty answering questions beyond a rudimentary level. | Presenters cannot answer questions posed by viewers for lack of knowledge on the topic. |
| **Sources:**  **Quantity**  **Format Quality** | All cited works in CSE format with no errors. Sources relevant and useful—number is optimal to support the thesis. | Some cited works in CSE format with some inconsistencies. Adequate number of sources. | Few cited works, CSE format with many errors. Minimal number of sources. | No citations, incorrect format or not enough sources. |
| **Mechanics:**  **Spelling**  **Grammar** | One or no spelling, punctuation, capitalization, grammar, or usage errors. | Two or three mechanics errors. | Four or five mechanics errors. | More than five mechanics errors, resulting in difficult reading. |
