# Supplementary material for: Thanks for inviting me to the party: Virtual poster sessions as a way to connect in a time of disconnection
Source: Ecol Evol. 2020 Sep 14;10(22):12423–30. doi: 10.1002/ece3.6756 (PMC7679537; doi:10.1002/ece3.6756)
Supplement: Supplementary file 2 — Appendix S2 [file ECE3-10-12423-s002.docx]

**Appendix S2. Virtual Poster Session Handout.**

This poster session highlights the research done by Ecology students during the latter half of this semester. The research questions addressed by each group of presenters revolve around the theme of anthropogenic influences on ecological systems. These students have put in a **ton** of effort into preparing these 16 posters, and deserve this chance to present their findings to a wide audience.

Please join us to view these posters, to learn something new, and to celebrate the work we’ve all endured for the past few weeks! While you are in the poster session please:

1. Feel free to interact with all the posters, and ask questions of any of the presenters.
2. Remember that this is a university function, and that people around you in this virtual space can hear you, so…
3. Please be courteous and respectful to the presenters and others around you.

Due to the ongoing health crisis, this poster session will be held within an online virtual reality (VR) space developed with Mozilla Hubs. Multiple ‘rooms’ will be set up with two or three posters in each room and links to two adjacent rooms. Each user will enter the system through a separate room (labelled ‘Atrium’ below) with links to each of the other rooms. Once in one of the presentation rooms, you can navigate in a loop through the six presentation rooms as diagramed in the schematic below.

Detailed information about navigating Hub’s virtual space can be found by clicking this [link](https://hubs.mozilla.com/docs/hubs-create-join-rooms.html#enter-the-room), and controls within Hubs can be found [here](https://hubs.mozilla.com/docs/hubs-controls.html).

As this software is browser-based, users should be able to join from any device with internet access (i.e. desktop, laptop, tablet, smart phone). Device-specific control instructions are available in the links above.

[Here, students were provided a Google Drive link where the Mozilla Hubs atrium link would be posted, as well as a reminder of the scheduled day and time of the session.]

To fully enter and engage with each of the rooms, you will need to follow these steps:

1. Click the provided link (or copy/paste the link into your web browser) to open the webpage. You will now be in what is called the ‘lobby’ for that room. You can see what is happening in the room, but you cannot interact with anything yet.
2. Click ‘Enter Room’. This will take you to a screen where you can select your avatar.
3. Delete the provided avatar name and type your name and affiliation. Please use a name we will all recognize you by and the class you are associated with. So the student presenters, for example, will look like: XXX-Rob W.
4. Choose an avatar to represent you:
   1. Click ‘Browse Avatars’
   2. Scroll through the list of avatars – there are multiple pages.
   3. Click an avatar to select it.
   4. Click ‘Accept’.
5. At this point, if you have a VR headset, you can choose to connect to that – if not, click ‘Enter on Screen’.
6. You will need to grant permissions for the website to use your microphone, you will need this capacity to interact with the presenters! – Click ‘Next’. (your browser may present you with a pop-up asking you to grant access to your microphone – click ‘Allow’/’Yes’/etc.)
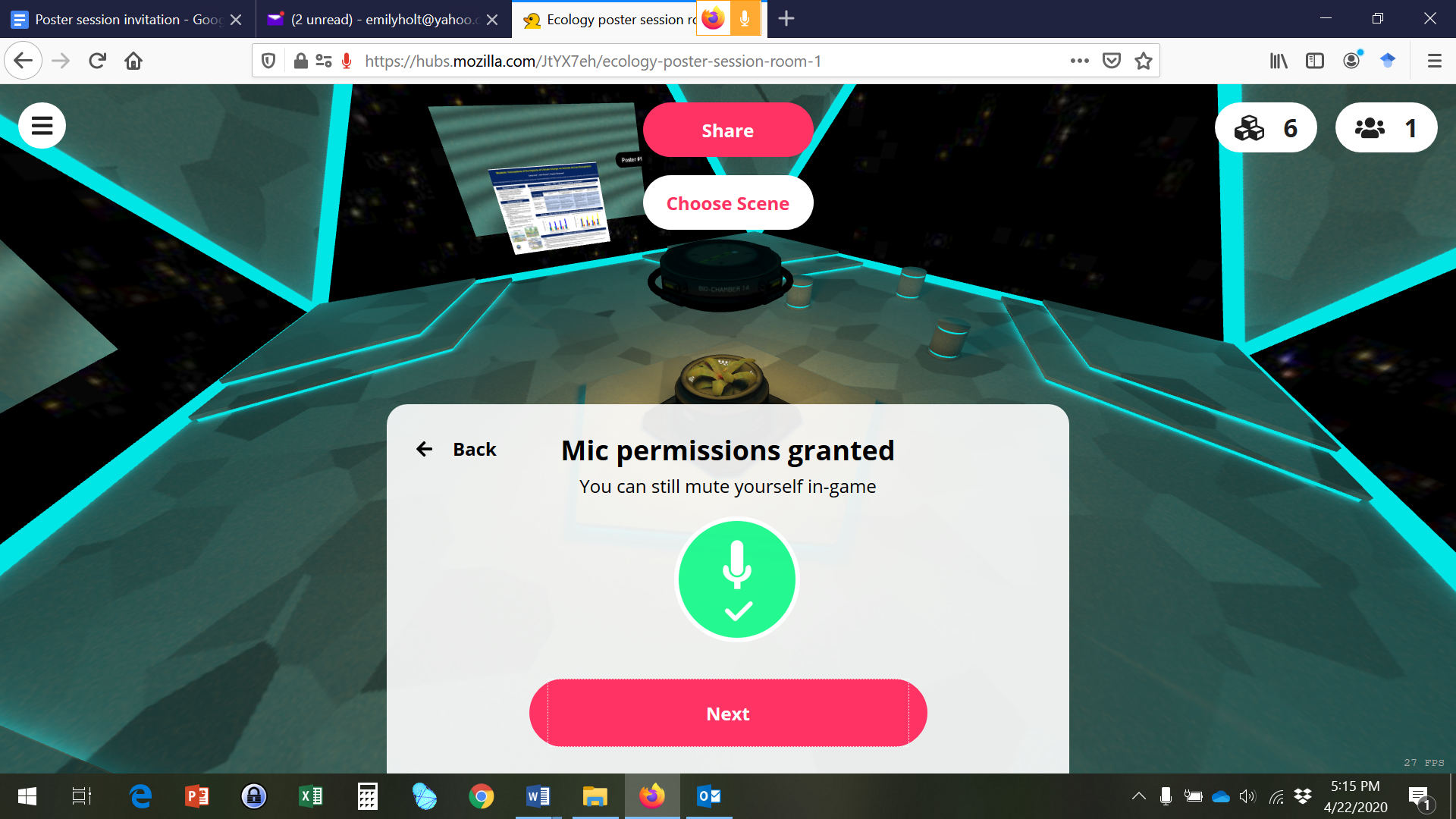
 shows that your microphone is working properly.
7. Click ‘Next’ after you have tested that your audio is working
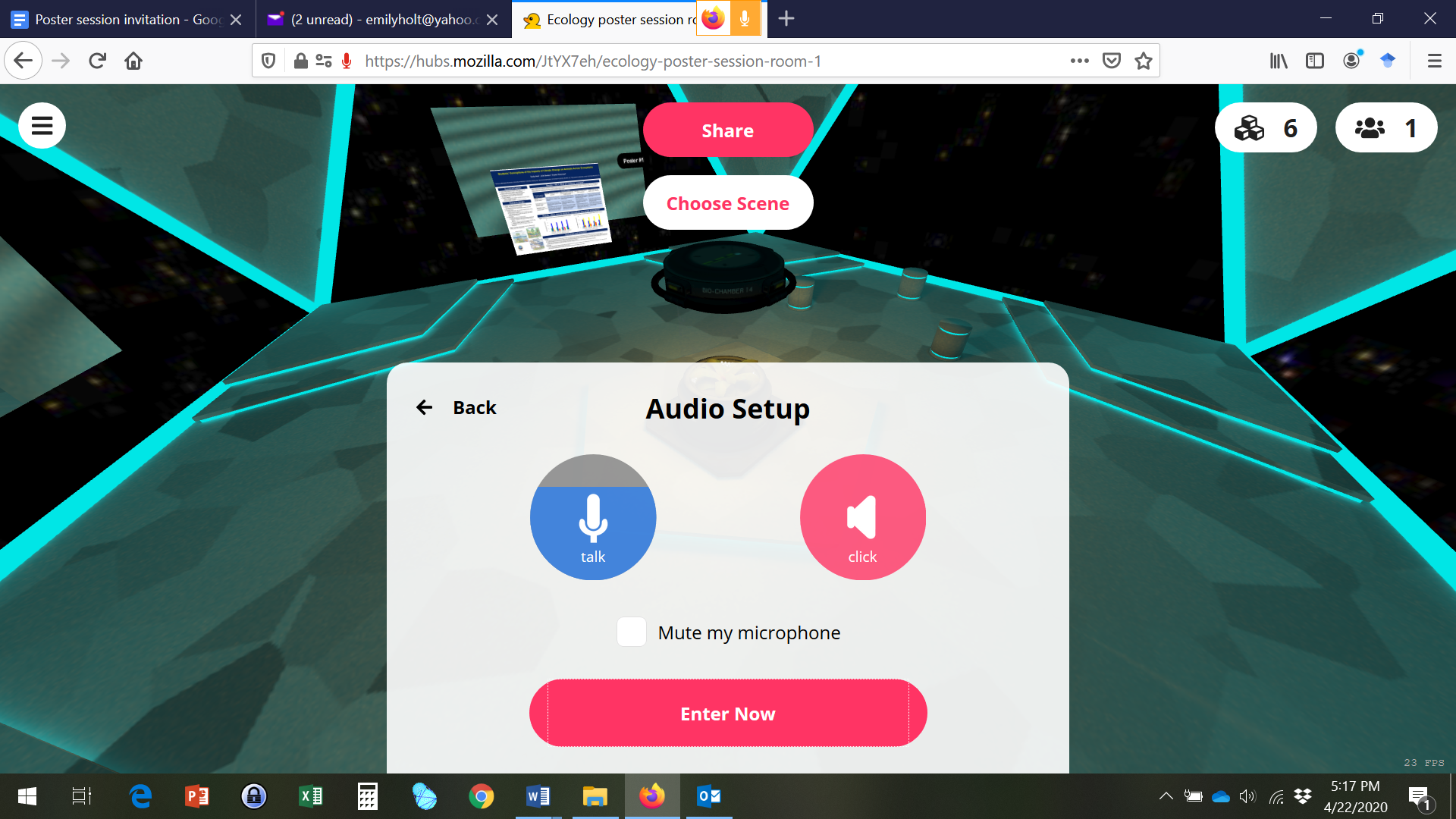

8. Click ‘Enter Now’.
9. You will hear a tone indicating you are now in the room.

When you first enter the ‘Atrium’ you will need to choose a presentation room to begin your participation in the poster session, so choose a room with only a few people. In the upper right corner of each room (and lobbies to rooms) is an indication of how many people they are currently holding
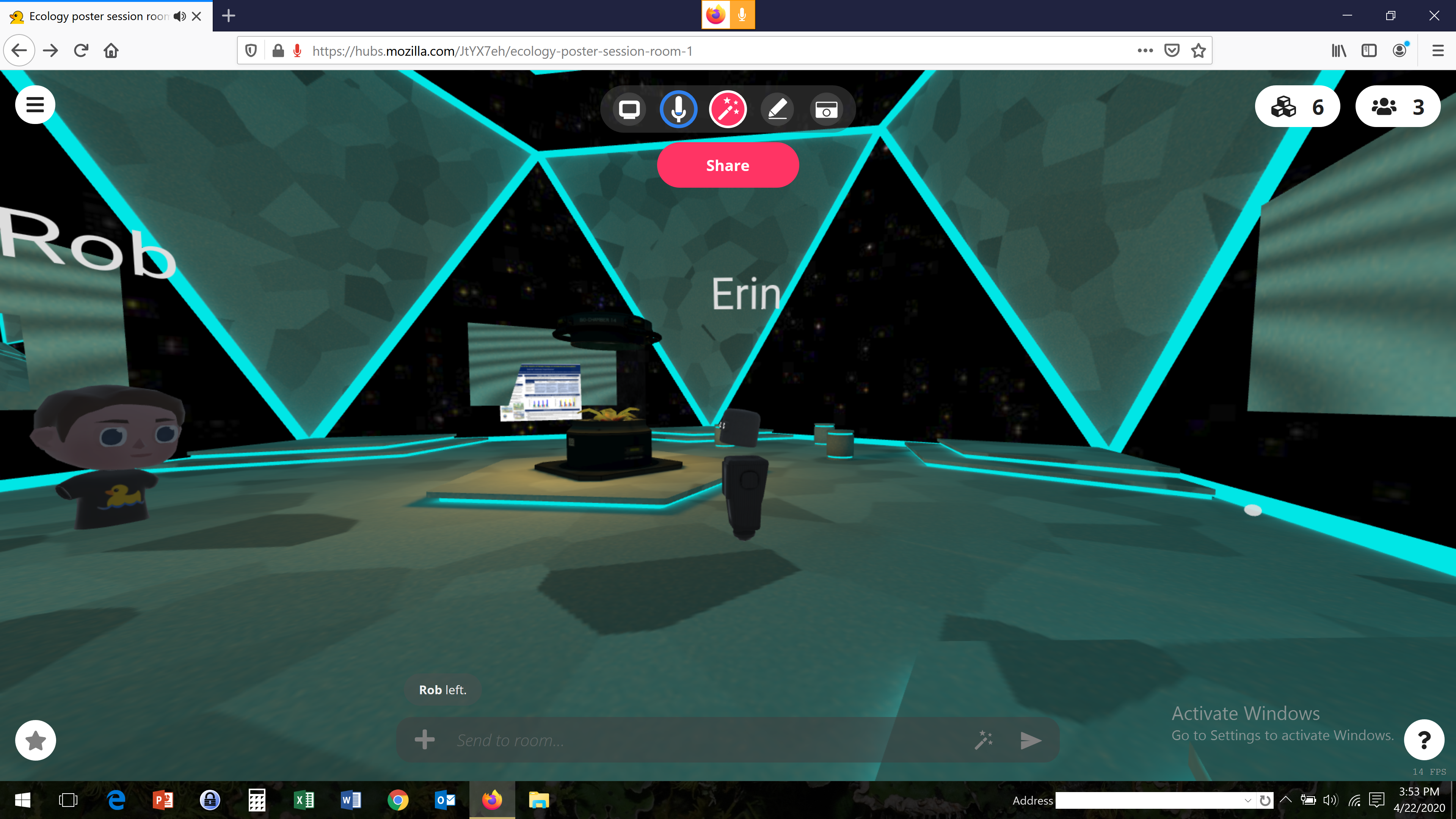
You will have to follow the above procedures for every presentation room you enter. Due to processing capacity, each room is limited to 24 users. If the room you are trying to enter is full, you will be placed in a holding pattern in the ‘lobby’. Once another person leaves the room, you will be allowed by the server to enter. Because of this, and because the ‘Atrium’ is the room that everyone will filter through when they enter this space, enjoy the posters and interactions in each given room but **please keep moving through rooms** after you have finished your discussions with presenters.

To move between rooms:

1. Approach one of the links in each room labeled with “Next Room” and “Previous Room”. The link will appear as an image of the lobby of the room to which it leads.
2. Click the ‘Open Link’ button that appears when you hover your cursor over the image.
3. Follow the above procedures to enter the new room (you shouldn’t need to choose a new avatar or name, just grant permission to use your microphone).

Some tips for a great experience:

1. We found that phones or computers (rather than tablets) work best in Hubs.
2. Unless you can find a quiet location to participate, we highly recommend headphones.
3. Mozilla Hubs can be a processing intensive program, so we recommend closing other background program (e.g. Zoom, YouTube) and have a charger nearby!
4. To easily access options within Hubs (e.g. changing other people’s volume, opening a link on a poster to make it full screen), hold the spacebar on a computer and double tap on a phone (to enter in or out of options mode).
5. To leave the poster session entirely when you are done viewing the posters, simply close your browser!

We invite you to practice using Mozilla Hubs before the session by testing out the Practice Rooms that we’ve created that will simulate the space for the actual poster session. Please test it out any time prior to the session and explore Hubs!
