# Supplementary material for: Thanks for inviting me to the party: Virtual poster sessions as a way to connect in a time of disconnection
Source: Ecol Evol. 2020 Sep 14;10(22):12423–30. doi: 10.1002/ece3.6756 (PMC7679537; doi:10.1002/ece3.6756)
Supplement: Supplementary file 3 — Appendix S3 [file ECE3-10-12423-s003.docx]

**Appendix S3. Reflection questions for instructors and external observers.**

1. What were your first impressions of the virtual ecology poster session?
2. From your point of view as an observer, what were some successes or benefits of the virtual ecology poster session?
3. From your point of view as an observer, what were some challenges of the virtual ecology poster session?
4. How did this virtual poster session compare to [past in-person BIO 360 or other course-based] poster sessions you have attended?
   1. Identify successes and challenges of each format.
5. What do you envision was the value of participating in this virtual poster session FOR STUDENTS?
6. What do you believe was the value of participating in this virtual poster session FOR YOU AS AN OBSERVER [as an educator or administrator]?
7. As an observer, did you interact with any student presenters during the session?
   1. If so, describe your interactions with these student presenters. Were there any notable differences in your interactions at this virtual poster session versus an in-person poster session environment? How do you think the student presenters adapted to this virtual environment?
8. Would you or your colleagues use a similar virtual format for future poster sessions in your classes? Why or why not?
9. What changes could be made to improve the effectiveness of this ecology virtual poster session in the future?
